# An Exploratory Study to Understand the Climate That Creates Opportunities (Ba) for Physiotherapists to Develop Professionally: A Workplace Climate That Enhances the Sense of Self-Growth

**DOI:** 10.7759/cureus.81593

**Published:** 2025-04-01

**Authors:** Koji Ikeda, Yasutomo Jono, Yoshiyuki Yoshikawa, Yuki Noda, Koji Takimoto, Ryo Mikami

**Affiliations:** 1 Division of Physical Therapy, Department of Rehabilitation, Faculty of Health Sciences, Naragakuen University, Nara, JPN

**Keywords:** climate factors, exploratory analysis, japan, physiotherapists, sense of self-growth, workplace

## Abstract

Objective: This study aimed to understand the climate that creates opportunities for physiotherapists to develop professionally by conducting an exploratory analysis of the workplace climate factors that enhance the sense of self-growth among them.

Methods: We recruited participants from March to May 2023 through physiotherapists’ networks and social media. Participants were sent a URL to access the study survey. A total of 357 Japanese physiotherapists who had provided their consent to participate in the survey engaged in the study. The questionnaire measured physiotherapists’ subjective perception of their current workplace across three aspects: a sense of self-growth, satisfaction level, and stress level. Further, they were asked to rate three aspects of their workplace: climate, human relationships, and the learning environment. The respondents were also asked to rate their subjective impression of the climate at their current workplace on 10 four-point scales, each with antonymous adjectives at the ends of the scale: (1) bright-dark, (2) exciting-boring, (3) warm-cold, (4) strict-loose, (5) honest-dishonest, (6) open-closed, (7) diligent-lazy, (8) passionate-apathetic, (9) cooperative-uncooperative, and (10) autonomous-heteronomous. Exploratory analysis was conducted to examine the relationship between each item and physiotherapists’ sense of self-growth. We formatted the scale responses as a 0-1 binary prior to analysis to minimize ambiguity in the response data. Binary logistic regression was performed, with the cutoff for statistical significance set at 5%.

Results: Sense of self-growth was significantly associated with a positive learning environment (β=0.41, OR=2.28, p<0.01). The learning environment was significantly associated with strictness (β=0.28, OR=1.89, p<0.05) and diligence (β=0.78, OR=4.90, p<0.01). The results suggest that a positive learning environment is essential for enhancing physiotherapists’ sense of self-growth; to create such an environment, fostering a climate that is perceived as strict and diligent is important.

Conclusions: A positive learning environment is essential for the development of physiotherapists’ sense of self-growth; to create such an environment, fostering a culture that is recognized as strict and diligent is crucial. Therefore, when developing workplace policies and training strategies, the following aspects must be considered: it is important to create a management and guidance system that fosters a workplace environment where it is easier to learn and that recognizes the importance of strictness and diligence in work.

## Introduction

In Japan, physiotherapists traditionally receive on-the-job training through a master’s apprentice system, where senior physiotherapists impart their knowledge and skills to junior physiotherapists. However, this system alone may no longer be adequate for on-the-job training, as frontline physiotherapists require more diverse and higher-level skills amid the diversification of values, increasing sophistication of healthcare, expansion in job roles, and increasing specialization [1,2]. Another issue is the shortage of leaders who can provide on-the-job training. This decline coincides with Japan’s changing social and healthcare landscape (e.g., the country’s work-style reform agenda and heightened concerns about workplace bullying), while the number of physiotherapists has rapidly increased [3].In the present study, we use the term “pro-growth occasions” (ba in Japanese) to refer to healthcare teamwork in frontline settings, healthcare workshops, rehabilitation settings, general workplace settings, and other settings in a physiotherapist’s working environment. In a previous study, we focused on pro-growth occasions for physiotherapists’ professional growth (“pro-growth occasions”) consisting of settings that encourage physiotherapists to engage in experiential learning and refine their skills [2]. Pro-growth occasions can emerge naturally or be designed and planned. Underpinning these occasions is an organizational culture or climate that is conducive to physiotherapist training. There is still no consensus on concepts such as “organizational culture,” “workplace norms,” and “workplace climate.” Exact interpretations and definitions of these concepts differ [4-10]. In this study, we use “climate” as a catch-all term for concepts such as workplace norms, climate, and organizational culture. In other words, we define “climate” as the sum of employees' perceptions of their workplace.

The concept of “climate” has been studied by scholars of industrial and organizational psychology [11,12]. In an analysis of Japanese companies, Yoshida and Takano [9] found that clearly codifying the company’s corporate climate as a company policy has a positive influence on job performance. Edmondson [13] analyzed the relationship between climate and growth, revealing that psychological safety facilitates learning and growth. The English literature on healthcare professionals has cited the climate as a causal factor in adverse healthcare workplace outcomes such as poor employee morale, employee stress, and increased accidents; burnout and staff turnover rates; and a high number of adverse events linked to issues in quality of care and performance issues [14]. A study focused on physical therapists and the organizational climate reported that physical therapist leaders contribute to an organizational climate of evidence-based physical therapy practice [15]. Further, the ethical climate of an organization is positively correlated with the job satisfaction of physical therapists [16]. Similarly, Japanese literature has linked the climate with burnout, turnover, stress, and satisfaction levels among medical and nursing care workers [17-22]. However, little is known about how the climate can facilitate physiotherapists’ professional growth. 

Japan has shown some interest in this question: In 2018, Japan revised the curriculum for physical therapy training institutions, adding content on physiotherapy management science, including content on talent development and organizational management [23]. However, the body of knowledge in physiotherapy management science comes largely from research in industrial and organizational psychology; few models and insights have been derived from the workplace experiences of Japanese physiotherapists [24]. Specifically, there is a lack of models or knowledge linking the creation of workplaces for physical therapists and human resource development. Therefore, there is a dearth of evidence on how a workplace and climate that facilitates physiotherapists’ professional growth can be created. Recent literature in other fields has highlighted the problem of a “loose workplace,” which has high employee turnover because it offers poor prospects for professional development [25]. Such studies suggest the need for a new approach to talent development. In this effort, we have been conducting research to support the growth of physiotherapists and have identified three work beliefs related to their growth [26]. However, research focused on the climate that promotes the growth of physiotherapists is lacking. Further, a climate that creates pro-growth occasions has not yet been identified. These gaps limit our ability to indirectly support the growth of physical therapists in creating a climate. If we could understand the climate that creates pro-growth occasions, managers could foster climates in their workplaces, teams, and frontline workflows that contribute to the development of pro-growth occasions, indirectly supporting on-the-job training. However, identifying pro-growth occasions and measuring the extent of a physiotherapist’s professional growth, when interpreted in many different ways, can be difficult. Therefore, this study defines a physiotherapist’s professional growth as a “sense of self-growth” and the workplace as an “occasion.” Using these definitions, we attempted to understand the climatic factors that enhance the physiotherapists’ sense of self-growth. In particular, there has been no study that focuses on the quality of the climate, structures it, and examines its relationship with growth. If these aspects become clear, it will be easier to create a climate that promotes growth in the workplace. This would also contribute to the creation of pro-growth occasions.

This study aimed to understand the climatic factors that enhance physiotherapists’ sense of self-growth to facilitate their professional development. We believe that this exploration will enable the development of a much-needed indirect, new approach to human resource development.

## Materials and methods

Participants

We recruited participants from March to May 2023 through physiotherapists’ networks and social media. They were sent a URL linked to the study survey. A total of 357 Japanese physiotherapists who had provided their consent to participate in the survey engaged in the study.

They communicated their informed consent by indicating that they had read and understood the written briefing provided in the first item of the questionnaire. The study was approved by the ethics committee of Naragakuen University (4-H033) and conformed to the Declaration of Helsinki.

Survey and measures

The survey was created using Google Forms (Appendix A1), and the URL was distributed. The survey questionnaire was pre-tested or validated by the researchers before full implementation. The questionnaire was kept anonymous and had four main parts: the first part was related to basic attributes: age, sex, years of clinical experience, and description of the workplace. The second part included three measures of the physiotherapist’s subjective perceptions of their current workplace, including a sense of self-growth, satisfaction level, and stress level. Each measure was rated on a four-point scale, with a higher score indicating a greater sense of self-growth and a higher satisfaction or stress level. The third part asked the respondents to rate three aspects of their workplace: climate, human relationships, and learning environment. Each aspect was rated on a four-point scale, with a higher score indicating a more favorable response. For the fourth part, the respondents were asked to rate their subjective impression of the climate at their current workplace on 10 four-point scales, each with antonymous adjectives at the ends of the scale: (1) bright-dark, (2) exciting-boring, (3) warm-cold, (4) strict-loose, (5) honest-dishonest, (6) open-closed, (7) diligent-lazy, (8) passionate-apathetic, (9) cooperative-uncooperative, and (10) autonomous-heteronomous. On each scale, a higher score indicated a more favorable response. In the case of bright-dark, for instance, 4 indicates “very bright” while 1 indicates “very dark.” These items are adjectives frequently used to describe the workplace climate in Japan. Participants were not allowed to complete the survey unless they answered all the questions to avoid missing data.

Analyses

Two analyses were performed. To minimize ambiguity in the response data, the scale responses were formatted as a 0-1 binary prior to analysis, with 1 representing a response of 4 or 3 and 0 representing a response of 2 or 1. While there is a possibility of information being lost in binary conversion, to effectively translate the survey results to the societal context, we considered a simple binary judgment to be practically effective. In addition, as how respondents make their choices varies according to their culture and personal biases, we aimed to increase data consistency by converting the four-level rating to a binary rating.

The first analysis consisted of a binary logistic regression in which the objective variables were the three measures of a physiotherapist’s subjective perception of their current workplace (sense of self-growth, satisfaction level, and stress level), and the three explanatory variables were the three workplace aspects (organizational climate, human relationships, and learning environment). The cutoff for statistical significance was set at 5%.

The second analysis consisted of a binary logistic regression in which the objective variables were the three workplace aspects (climate, human relationships, and learning environment) and the explanatory variables were the 10 perceptual categories of organizational climate. The cutoff value for statistical significance was set at 5%. Statistical analysis was performed using the Bell Curve for Excel (Social Survey Research Information Co., Ltd.).

## Results

The sample attributes were as follows (Table 1): 263 (73.7%) respondents were men, 91 (25.5%) were women, and three (0.8%) did not disclose their gender. The mean age was 35.3 ± 8.2 (mean ± standard deviation) years. The mean number of years of clinical experience was 12.1 ± 7.3 (mean ± standard deviation) years. Among medical workplaces, 81 (22.7%) respondents worked in acute medical care, 88 (24.6%) in recovery, and 29 (8.1%) in convalescence. Regarding long-term care workplaces, 61 (17.1%) respondents worked in home care (or daycare) services, and 18 (5.0%) worked in a care home. Additionally, 46 (12.9%) respondents worked in an educational or research workplace, and 34 (9.5%) worked in other workplaces.

**Table 1:** Sample attributes Gender: number and percentage; age, years of clinical experience: mean and standard deviation (SD); workplace: number and percentage

Questionnaire items	Overall n=357	
Gender (numbers)	n	%
Male	263	73.7
Female	91	25.5
No response	3	0.8
Number of years	mean	SD
Age (years)	35.3	8.2
Years of clinical experience as a physiotherapist (years)	12.1	7.3
Type of workplace (n, %)	n	%
Medical institution: acute medical care	81	22.7
Medical institution: recovery	88	24.6
Medical institution: convalescence	29	8.1
Long-term care: home-care, daycare	61	17.1
Long-term care: care home	18	5
Education or research institution	46	12.9
Other	34	9.5

Table 2 presents the results of the initial analyses.

**Table 2:** Associations of sense of self-growth, satisfaction, and stress with climate, human relationships, and the learning environment Analysis: a binary logistic regression, β: standardized partial regression coefficient, OR (95%CI): odds ratio (95% confidence interval), *: p < 0.05, **: p < 0.01

Sense of Self-Growth, Satisfaction, Stress					
Sense of self-growth					
Variables	β	OR (95%CI)		Wald (X^2^)	p-value
Climate	0.15	1.45 (0.68-3.1)		0.90	0.34
Human relationships	0.27	2.00 (0.91-4.37)		2.99	0.08
Learning environment	0.41	2.28 (1.4-3.73)		10.95	< 0.01**
Constant term		0.61 (0.36-1.03)		3.43	0.06
Satisfaction					
Variable	β		OR (95%CI)	Wald (X^2^)	p-value
Climate	0.80		6.87 (2.96-15.95)	20.07	< 0.01**
Human relationships	0.60		4.62 (1.83-11.69)	10.47	< 0.01**
Learning environment	0.70		4.13 (2.34-7.26)	24.16	< 0.01**
Constant term			0.06 (0.03-0.14)	42.82	< 0.01**
Stress					
Variable	β		OR (95%CI)	Wald (X^2^)	p-value
Climate	-0.42		0.36 (0.16-0.85)	5.42	0.02 *
Human relationships	-0.58		0.22 (0.09-0.61)	8.77	< 0.01**
Learning environment	-0.16		0.72 (0.45-1.16)	1.80	0.18
Constant term			12.32 (5.28-28.78)	33.72	< 0.01**

The sense of self-growth was significantly associated with a positive learning environment (β=0.41, OR=2.28, p<0.01). The coefficient of determination (R2) was 0.07; the regression equation was significant (p<0.01). The percentage of correct classifications was 68.4%.

Satisfaction was significantly associated with a positive climate (β=0.80, OR=6.87, p<0.01), positive human relationships (β=0.60, OR=4.62, p<0.01), and a positive learning environment (β=0.70, OR=4.13, p<0.01). The coefficient of determination (R2) was 0.32; the regression equation was significant (p<0.01). The percentage of correct classifications was 81.2%.

Stress was significantly associated with a positive climate (β=-0.42, OR=0.36, p<0.05) and positive human relationships (β=-0.58, OR=0.22, p<0.01). The coefficient of determination (R2) was 0.11; the regression equation was significant (p<0.01). The percentage of correct classifications was 63.9%.

Table 3 presents the results of the second analysis.

**Table 3:** Associations of climate, human relationships, and the learning environment with the 10 adjective pairs of climate Analysis: a binary logistic regression, β: standardized partial regression coefficient, OR (95%CI): odds ratio (95% confidence interval), *: p < 0.05, **: p < 0.01

Climate, Human Relationships, Learning Environment				
Climate				
Variables	β	OR (95%CI)	Wald (X^2^)	p-value
Brightness	0.91	8.85 (3.52-22.23)	21.49	< 0.01**
Excitement	0.09	1.22 (0.49-3.03)	0.18	0.67
Warmth	0.4	2.47 (0.98-6.2)	3.71	0.05
Strictness	-0.16	0.69 (0.25-1.92)	0.50	0.48
Honesty	-0.26	0.55 (0.19-1.59)	1.20	0.27
Openness	0.59	3.38 (1.32-8.67)	6.40	0.01*
Diligence	0.66	3.82 (1.31-11.14)	6.04	0.01*
Passion	0.17	1.41 (0.54-3.66)	0.50	0.48
Cooperativeness	0.58	3.53 (1.35-9.24)	6.63	0.01*
Autonomy	0.25	1.67 (0.75-3.74)	1.60	0.21
Constant term		0.09 (0.04-0.21)	31.07	< 0.01**
Human relationships				
Variable	β	OR (95%CI)	Wald (X^2^)	p-value
Brightness	0.5	3.3 (1.34-8.13)	6.77	< 0.01**
Excitement	0.66	4.06 (1.58-10.4)	8.50	< 0.01**
Warmth	0.69	4.86 (1.86-12.68)	10.41	< 0.01**
Strictness	-0.36	0.44 (0.17-1.17)	2.68	0.1
Honesty	-0.35	0.46 (0.15-1.43)	1.79	0.18
Openness	0.3	1.86 (0.68-5.1)	1.46	0.23
Diligence	0.49	2.68 (0.92-7.8)	3.25	0.07
Passion	-0.25	0.61 (0.22-1.67)	0.94	0.33
Cooperativeness	0.75	5.12 (1.84-14.25)	9.78	< 0.01**
Autonomy	-0.09	0.82 (0.36-1.87)	0.22	0.64
Constant term		0.29 (0.14-0.59)	11.77	< 0.01**
Learning environment				
Variable	β	OR (95%CI)	Wald (X^2^)	p-value
Brightness	0.09	1.24 (0.55-2.79)	0.28	0.6
Excitement	0.25	1.69 (0.87-3.29)	2.39	0.12
Warmth	-0.07	0.85 (0.38-1.87)	0.17	0.68
Strictness	0.28	1.89 (1.02-3.5)	4.14	0.04*
Honesty	-0.03	0.93 (0.44-1.96)	0.04	0.85
Openness	0.09	1.2 (0.61-2.34)	0.28	0.59
Diligence	0.78	4.9 (2.65-9.07)	25.65	< 0.01**
Passion	0.17	1.4 (0.73-2.69)	1.03	0.31
Cooperativeness	0.18	1.49 (0.69-3.19)	1.04	0.31
Autonomy	-0.13	0.77 (0.44-1.35)	0.84	0.36
Constant term		0.23 (0.12-0.43)	21.03	< 0.01**

Climate was significantly associated with brightness (β=0.91, OR=8.85, p<0.01), openness (β=0.59, OR=3.38, p<0.05), diligence (β=0.66, OR=3.82, p<0.05), and cooperativeness (β=0.58, OR=3.53, p<0.05). The coefficient of determination (R2) was 0.53; the regression equation was significant (p<0.01). The percentage of correct classifications was 89.6%.

Human relationships were significantly associated with brightness (β=0.50, OR=3.30, p<0.01), excitement (β=0.66, OR=4.06, p<0.01), warmth (β=0.69, OR=4.86, p<0.01), and cooperativeness (β=0.75, OR=5.12, p<0.01). The coefficient of determination (R2) was 0.48; the regression equation was significant (p<0.01). The percentage of correct classifications was 89.4%.

Learning environment was significantly associated with strictness (β=0.28, OR=1.89, p<0.05) and diligence (β=0.78, OR=4.90, p<0.01). The coefficient of determination (R2) was 0.21; the regression equation was significant (p<0.01). The percentage of correct classifications was 75.6%.

## Discussion

This study aimed to understand the climatic factors that enhance physiotherapists’ sense of self-growth to facilitate their professional development. Our first analysis revealed that self-growth is positively associated with the workplace learning environment, implying that physiotherapists are more likely to experience a sense of self-growth if the workplace is designed in a way that makes them feel that it is a good learning environment. Similarly, satisfaction was associated with climate, the learning environment, and human relationships, whereas stress levels were associated with climate and human relationships.

These results imply that physiotherapists are more likely to experience job satisfaction if the workplace features a positive climate, a positive learning environment, and positive human relationships. Further, they are likely to experience lower stress levels if the workplace has a positive climate and positive human relationships. One possible strategy to ensure that physiotherapists perceive the learning environment as positive involves providing tangible amenities, such as libraries and training facilities. However, this strategy may not be feasible in certain workplaces. A more feasible approach might be to provide intangible infrastructure such as lunchtime seminars and brief consultations with senior physiotherapists. Further research on these intangible measures is required. However, it must be noted that the coefficient of determination for the regression equation is low, and the percentages of correct classifications are only 68% (sense of self-growth), 81.2% (satisfaction), and 63.9% (stress), thus limiting our interpretations of the results.

The second analysis focused on the relationship between climate, human relationships, and the learning environment with the perceptual categories (qualitative) of organizational climate. As well as being measured contextually, organizational climate is sometimes rated in perceptual language (e.g., “bright,” “fun”). In other research fields, perceptual terms, such as the comfort of a building, the environment, or a company’s image, have been used to measure climate [27]. We believe that understanding the climate using these terms could make it easier to indicate the direction for creating the same.

The main results are as follows. We found that a positive organizational climate was associated with the perceptual categories of brightness, openness, diligence, and cooperativeness; as such, physiotherapists are more likely to perceive the climate as positive if they perceive it as bright, open, diligent, and cooperative. Positive human relationships were associated with brightness, excitement, warmth, and cooperativeness, implying that physiotherapists are more likely to perceive their human relationships as positive if they perceive the climate as bright, exciting, warm, or cooperative. Meanwhile, a positive learning environment was associated with strictness and diligence. Therefore, fostering a climate aligned with these aspects could contribute to creating a positive workplace for physical therapists. However, the coefficients of determination for the regression equation were 0.53 (climate), 0.48 (human relationships), and 0.21 (learning environment), and the percentages of correct classifications were 89.6% (climate), 81.2% (human relationships), and 63.9% (learning environment); thus, caution is required when interpreting the results.

On the premise that professionalism involves having the right beliefs about work, Matsuo [28,29] suggested that having the right work beliefs contributes to growth, in that it facilitates experiential learning and sustains learning over the long term. Matsuo also reported that such work beliefs are formed in a cooperative climate characterized by free and open dialogue. Ikeda et al. [26] identified three categories of work beliefs held by Japanese physiotherapists: those related to building professional relationships, those related to engaging in broad practices with a sense of professional pride, and those related to treatment outcomes. However, to the best of our knowledge, the literature presents no findings on the type of climate that facilitates these three categories of work beliefs nor on the type of climate that facilitates physiotherapists’ professional growth. Therefore, it remains unclear how climate facilitates or otherwise influences professional growth among physiotherapists working in Japan.

However, the current results suggest that physical therapists perceived the learning environment at work positively when they felt a sense of personal growth and that they perceived the learning environment positively when they perceived strictness and diligence. We believe that this climate fosters the beliefs of Japanese physiotherapists and contributes to their professional growth. Further, we expect that this contributes to the creation of pro-growth occasions. However, care should be taken, as being too strict or hardworking can lead to increased stress. If we can intentionally create such an environment, we can expect physical therapists' learning to be naturally promoted and their growth to be supported.

The mechanism for enhancing the sense of self-growth might differ from that for mitigating stress, given our observation that the sense of self-growth was associated with the learning environment, whereas stress was associated with climate and human relationships. Furuya [25] noted that loose workplaces have a high turnover of young employees because they lack a sense of self-growth or find the work too stressful; therefore, these two issues must be addressed as a part of talent development and turnover prevention strategies. The problem of loose workplaces that Furuya [25] identified may occur in physiotherapist workplaces too, given our observation that the mechanism for a sense of self-growth differs from that for stress. We observed an association between satisfaction and all three variables (climate, human relationships, and the learning environment), implying that physiotherapists will feel more satisfied with their jobs if their workplace is designed with a focus on enhancing their sense of self-growth and mitigating stress.

Our findings offer insights for employers. We observed that a positive learning environment is essential for physiotherapists to experience a sense of self-growth; therefore, employers should consider strategies to foster a workplace that employees perceive as strict and diligent. By developing an organizational culture with these points in mind, employers can indirectly enhance physiotherapists’ sense of self-growth. Moreover, at a time when on-the-job training for junior employees has become increasingly “loose” amid excessive concerns about workplace bullying, our results suggest that it is essential to provide on-the-job training that employees perceive as strict and diligent. Specifically, this would help create a positive organizational climate through efforts such as conducting training to develop strict and diligent leaders, holding technical training sessions at facilities, and encouraging active academic activities. Field and Abelson [30] delineated three climate focuses: (1) external (physical environment, sociocultural environment); (2) organizational (structures, rules, technologies); and (3) individual (managers’ behavior and leadership patterns). However, further research is necessary to understand the construct of physiotherapists’ workplace perceptions and how climate shapes such perceptions.

Lastly, while this study analyzed overall trends in Japan, it is possible that trends may differ depending on gender, age, years of clinical experience, and workplace characteristics. Furthermore, this study only examined the correlations between factors and did not clarify causal relationships; these are aspects to be addressed in future research. Additionally, considering factors related to history, culture, and institutional design, it is possible that the results of this study can only be applied to the Japanese context; making the knowledge applicable globally is a future research challenge.

The limitations of this study are as follows. This study used cross-sectional convenience sampling, which is not representative of the general population, and self-reported data, which could lead to biases such as social desirability bias or recall bias. Therefore, care must be taken during interpretation. We analyzed only the sense of self-growth, satisfaction, stress, climate, human relationships, and learning environment, along with the 10 perceptual categories of climate/image (quality), indicating that the roles of other factors remain unclear. Thus, no definitive conclusions can be drawn from the results. In the future, the sampling method may have to be changed or other factors added to the survey. Another limitation is that a sense of self-growth does not necessarily equate to professional competence. Therefore, our analysis did not cover the extent to which the employees developed competencies. While the sense of self-growth is, in a broad sense, an aspect of professional growth, it may be problematic to apply our observations to the sense of self-growth in actual on-the-job training. Therefore, factors that indicate growth other than a sense of personal growth must be investigated. Further research is needed to understand the construct of an organizational climate in which physiotherapists work and how such a climate shapes their experiential learning.

## Conclusions

This study suggests that a positive learning environment is essential for enhancing physiotherapists’ sense of self-growth. In creating such an environment, it is important to foster a climate that is perceived as strict and diligent. If we can intentionally create such an environment, we can expect improvements in physical therapists' learning, supporting their growth. Future studies must change the sampling method or add other factors to the survey and investigate variables that indicate growth other than a sense of personal growth.

## Appendices

**Table 4:** Questionnaire

Content of the Question
1) How old are you?
2) Please describe your gender. [Male Female No response]
3) For how many years have you worked as a physiotherapist?
4) Please describe your current workplace.
a) Medical institution: acute medical care
b) Medical institution: recovery
c) Medical institution: convalescence
d) Long-term care: home care, daycare
e) Long-term care: care home
f) Education or research institution
g) Other
5) To what extent have you grown in your current workplace? Rate your growth on a four-point scale.
I’ve stagnated 1 ・ 2 ・ 3 ・ 4 I’ve grown
6) Rate your satisfaction with your current workplace on a four-point scale.
I’m unsatisfied 1 ・ 2 ・ 3 ・ 4 I’m satisfied
7) Rate the stressfulness of your workplace on a four-point scale.
No stress 1 ・ 2 ・ 3 ・ 4 Very stressful
8) How do you feel about your current workplace?
a) Climate: Bad (1 point) Not good (2 points) Not bad (3 points) Good (4 points)
b) Human relationships: Bad (1 point) Not good (2 points) Not bad (3 points) Good (4 points)
c) Learning environment: Bad (1 point) Not good (2 points) Not bad (3 points) Good (4 points)
9) Describe your subjective impressions of the organizational climate of your current workplace. Please respond spontaneously, without thinking too much.
1 Dark 1 ・2 ・3 ・4 Bright
2 Boring 1 ・2 ・3 ・4 Exciting
3 Cold 1 ・2 ・3 ・4 Warm
4 Loose 1 ・2 ・3 ・4 Strict
5 Dishonest 1 ・2 ・3 ・4 Honest
6 Closed 1 2 3 4 Open
7 Lazy 1 ・2 ・3 ・4 Diligent
8 Apathetic 1 2 3 4 Passionate
9 Uncooperative 1 2 3 4 Cooperative
10 Heteronomous 1 2 3 4 Autonomous
